# Vision-Based Pose Estimation for Robot-Mediated Hand Telerehabilitation

**DOI:** 10.3390/s16020208

**Published:** 2016-02-05

**Authors:** Giuseppe Airò Farulla, Daniele Pianu, Marco Cempini, Mario Cortese, Ludovico O. Russo, Marco Indaco, Roberto Nerino, Antonio Chimienti, Calogero M. Oddo, Nicola Vitiello

**Affiliations:** 1 Department of Control and Computer Engineering, Politecnico di Torino, Corso Duca degli Abruzzi 24, Turin 10129, Italy; ludovico.russo@polito.it (L.O.R.); marco.indaco@polito.it (M.I.); 2 Institute of Electronics, Computer and Telecommunication Engineering, National Research Council, Corso Duca degli Abruzzi 24, Turin 10129, Italy; daniele.pianu@ieiit.cnr.it (D.P.); roberto.nerino@ieiit.cnr.it (R.N.); antonio.chimienti@ieiit.cnr.it (A.C.); 3 The BioRobotics Institute, Scuola Superiore Sant’Anna, viale Rinaldo Piaggio 34, Pontedera 56025, Italy; m.cempini@sssup.it (M.Ce.); m.cortese@sssup.it (M.Co.); oddoc@sssup.it (C.M.O.); n.vitiello@sssup.it (N.V.)

**Keywords:** hand telerehabilitation, hand exoskeleton, motion tracking, upper limb rehabilitation

## Abstract

Vision-based Pose Estimation (VPE) represents a non-invasive solution to allow a smooth and natural interaction between a human user and a robotic system, without requiring complex calibration procedures. Moreover, VPE interfaces are gaining momentum as they are highly intuitive, such that they can be used from untrained personnel (e.g., a generic caregiver) even in delicate tasks as rehabilitation exercises. In this paper, we present a novel master–slave setup for hand telerehabilitation with an intuitive and simple interface for remote control of a wearable hand exoskeleton, named HX. While performing rehabilitative exercises, the master unit evaluates the 3D position of a human operator’s hand joints in real-time using only a RGB-D camera, and commands remotely the slave exoskeleton. Within the slave unit, the exoskeleton replicates hand movements and an external grip sensor records interaction forces, that are fed back to the operator-therapist, allowing a direct real-time assessment of the rehabilitative task. Experimental data collected with an operator and six volunteers are provided to show the feasibility of the proposed system and its performances. The results demonstrate that, leveraging on our system, the operator was able to directly control volunteers’ hands movements.

## 1. Introduction

Traditional rehabilitation is performed in a one-to-one fashion, namely one therapist (or sometimes several) working with one patient, leading to high personnel and management costs, especially for demanding patients such as those with brain or post surgery injuries. Due to the high hospitalization costs, all these patients are leaving clinics and returning to their homes sooner than in the past [1], when their rehabilitative program is not yet finished. These patients can greatly benefit from a telerehabilitation equipment, which is able to provide remote assistance and relief without the burden of going to the clinic on a daily basis. On the other hand, therapists can surely benefit from non-invasive systems capable of acquiring information about their movements which are then sent to the patient (or even to many patients), possibly in real-time to allow a direct control; modern vision-based techniques offer interesting sparks in such way. The possibility to provide high quality rehabilitation programs regardless of patients physical location and leveraging on vision is thus certainly attractive.

### 1.1. Telerehabilitation

Typical telerehabilitation systems require patients to perform exercises following instructions given from a domestic PC, often in form of a Virtual Reality (VR) environment or a video game, while their motion kinematics is captured through sensorized tools such as gloves and grippers for off-line evaluation from the operator. Relevant examples are the computer-based biomechanical evaluation tools Eval by Greenleaf Medical (Portola Valley, CA, USA) [2], and the system proposed by Popescu *et al.* [3,4]. This last system comprises a VR environment, the force feedback glove “Rutgers Masters”, and a series of networked PCs: one at patient’s home, the others recording the rehabilitation performance in the clinical facility. Authors in [5] proposed a sensorized commercial hand glove, namely the HumanGlove, produced by Humanware s.r.l. (Pisa, Italy), for functional assessment of both the hand and the fingers. Despite being proof of the telerehabilitation relevance, these systems cannot provide feedback on the mobilization of the impaired articulations.

Different is instead the case of the telerehabilitation systems which establish Real Time (RT) direct links between the operator and the patient. An example is the system by Holden *et al.* [6,7,8], where a training-by-imitation rehabilitation strategy is enforced through a virtual avatar on the patient’s screen and the supervision of an operator. A drawback of such system is that it does not allow a direct intervention on subject movements but only corrections on the teaching unit.

Recent studies have suggested that for upper limb functional recovery, repetitive and long duration rehabilitation using robots is helpful [9,10,11,12,13,14,15,16]. This becomes evident especially when the robot system can be directly controlled by the operator, who can tune the therapy to the current patient’s conditions and actual residual abilities. Many studies dealt with the development of systems providing at-home rehabilitation therapy through robotic devices, without sacrificing quality of care: such systems are addressed as *master–slave* setups. Master–slave setups have been introduced also in mission-critical environments, such as in the field of tele-surgery [17]. The master unit records the intended motion from the operator, who guides the patient along a desired motion pattern, adjusting the task parameters as soon as needed on the basis of the feedbacks received by the slave unit.

The system for the home-treatment of elbow hypertonia presented by Peng *et al.*, in [18], and the system for upper-limb function recovery by Duong *et al.* [19] are examples of valuable *master–slave* setups. In an earlier application of the hand exoskeleton (HX) [20], the master units consists of the commercial sensorized glove Acceleglove (AnthroTronix, Silver Spring, MD, USA), worn by the operator and tracking his movements, and of a post-processing custom Java routine, providing RT records of the intended rehabilitation exercises.

### 1.2. Vision-Based Hand Pose Estimation

Despite being a natural option for mastering hand telerehabilitation, glove-based interfaces are typically expensive and may entangle therapist’ movements, thus compromising the efficacy of the protocol. In addition, they typically require calibration prior to each usage. A valid alternative comes from modern motion tracking technologies, which instead offers many advantages in terms of usability, reduced costs and learning time, and do not require calibration procedures. In this field, the KiReS (Kinect Rehabilitation System) [21] is a full-body telerehabilitation system based on Kinect, which implements a markerless video tracker of user movements. While performing the exercises, users are shown two 3D avatars: one is a 3D representative of correct movements to follow and one represents the user and its movements as captured by the Kinect. Markerless video tracking is a viable solution for master units in a master–slave setup too, as the operator can perform an exercise in front of the camera, while a robotic device guides the patient through its correct execution.

We propose a novel paradigm based on modern Vision-based Pose Estimation (VPE) and Hand Tracking techniques. VPE has played a leading role in the field of Human Robot Interaction (HRI), and has already demonstrated its applicability to remote control of robotic actuators [22]. With the availability of consumer grade RGB-D (RGB-Depth) sensors, VPE algorithms have gained momentum. State-of-the art solutions based on RGB-D cameras [23,24,25] for real-time full-body or hand tracking and pose estimation achieve impressive results. Moreover, VPE interfaces are intuitive enough to be used even from untrained personnel (e.g., a generic caregiver) [26].

In the context of pose estimation using RGB-D sensors (more generally, within the field of VPE), we can distinguish between two main approaches: *model-based* (also known as *generative*) and *appearance-based* (also called *discriminative*) ones.

Algorithms following the model-based approach search, within the space of possible hand poses, the one which minimizes a *dissimilarity* function with respect to the hand as seen by the RGB-D sensor. This research is often expressed as a non-linear optimization problem. Particle Swarm Optimization (PSO) [27] is a well-established algorithm specifically developed to optimize continuous non-linear functions. It is commonly employed in model-based approaches [25] to guarantee the convergence process with reasonable timing. Nevertheless, these approaches show limitations in reaching RT performances on consumer hardware.

Appearance-based approaches rely on machine learning algorithms specifically trained to estimate hand poses from run-time observations. Here the training represents the most demanding computational task, but it is performed only once and off-line. These approaches thus easily achieve RT performances. A previous study by Shotton *et al.* [23] lays the foundations for current state-of-the-art: authors perform a per-part classification of the human body using a Random Forest (RF) classifier [28] and simple per-pixel features which are computed on data acquired from a Kinect sensor. Human body parts are then clustered to approximate skeleton joints. Keskin *et al.* [24] applied successfully the same approach to the hand, which is segmented from the rest of the body, divided into parts and clustered to approximate its joints.

### 1.3. Vision-Based Hand Telerehabilitation

Currently, only few works address master–slave hand telerehabilitation, and very few leverage on vision-based techniques. To the best of our knowledge, no master–slave platform for hand tele-rehabilitation based on RGB-D sensors and vision-based algorithms has been presented. In this paper, we introduce a mechatronic master–slave setup for RT hand telerehabilitation, exploiting partial results from [20], but with the master guidance based on VPE hand tracking algorithm. The proposed setup combines three independent subsystems, enabling important features for a telerehabilitation protocol: (i) a VPE system through which the operator is able to dynamically drive patients’ hands along a desired exercise; (ii) the multi-joints robotic hand exoskeleton HX [29], driving the subject’s hand, and posed under direct control of the operator; (iii) a sensorized graspable object [30], which detects the fingertip grasp force during the manipulation exercises and further feeds it back it to the operator. The operator receives as additional feedback the measured positions from the HX joints, to reliably assess quality and percentage of completion of the exercises.

We argue that the usage of VPE-based master–slave system is attractive, since these systems provide the following advantages: (i) reduced costs and stress for the patients without compromising quality and accuracy of the rehabilitation; (ii) reduced discomfort and time occupation for the therapist, whom hand movements are not entangled and can be freely shaped but also recorded for being later sent to the slave interface, thus ensuring that a patient is performing several time exactly the same exercise or that different patients are following the same therapy; (iii) measurable and precise updates about patients’ performances provided to the operator, who can tune the rehabilitation therapy on the needs and behaviors of any single patient.

The main aim of this work is to demonstrate through an early validation stage that vision-based robot-mediated hand telerehabilitation is actually feasible. Experimental results achieved with an operator and six healthy volunteers prove the overall feasibility of our system, and the stability of the VPE-based telerehabilitation setup across different speed settings. In addition, experiments show that no user had difficulties nor discomfort in wearing the exoskeleton and performing the exercises and that the operator always had a direct RT control over their movements.

The remaining of the paper is organized as follows: Section 2 discusses the theoretical approach and practical implementation of our solution, as well as the integrated technologies description; Section 3 discusses results derived from our experiments; and finally Section 4 concludes the paper and presents planned future activities.

## 2. Experimental Section

### 2.1. System Overview

This Section introduces the master–slave telerehabilitation mechatronic apparatus, composed of three main subsystems: (i) the master unit, which consists of a consumer RGB-D camera and a VPE algorithm; the slave unit, which consists of (ii) a powered hand robotic exoskeleton; and of (iii) a sensorized object, recording gripping forces when handled. Master and slave units are connected by means of a bidirectional communication link. The master unit records, processes and conveys information about the operator’s hand, and sends RT motion commands to the slave unit, which mobilizes patient’s hand. The patient can grasp the sensorized object with his hand moved by the robotic exoskeleton. The master unit receives both pieces of feedback from the robotic exoskeleton and recordings of the detected grasping forces.

#### 2.1.1. Master Unit

Here, we propose a custom implementation of the per-part hand classification framework presented in [24], adapting it to our hand telerehabilitation task. Next, paragraphs briefly introduce the RF classifier and present our custom implementation.

##### Random Forests

Random Forests [28] are an ensemble of decision trees classifiers trained on a random subset of features and training data. Intermediate nodes store a feature-threshold pair $\left(F,\tau \right)$ learned during the training phase. Starting from the root node, for each input datum x, the feature response $F\left(x\right)$ is compared to the threshold *τ*; the datum is then forwarded to one of the child nodes according to the comparison result. Given a tree *T*, the comparison is repeated until a leaf node is reached, where a probability distribution $P_{T}\left(c|x\right)$ over all possible classes C is stored. The datum x descends every tree in the forest and the final probability distribution P(c|x) is given by the average of the $P_{T}\left(c|x\right)$ of the reached leaves.

**Figure 1:**
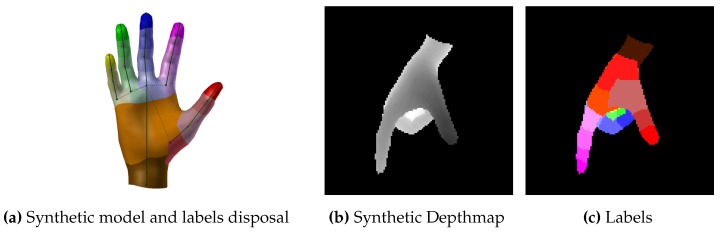
Model (**a**) and input data (**b**,**c**) used for training the RF classifier.

In the context of per-part hand classification, the input for the RF is represented by the pixels of the depthmaps as acquired from the RGB-D camera. For each pixel, the RF per-class posterior represents the probability that it belong to a given hand part. We distinguish between 22 different hand parts, centered with respect to finger joints, fingertips, wrist and palm center (see Figure 1a).

##### Features

The same feature presented in [23] is computed per-pixel during both RF training and at run-time. More specifically, given a depthmap *D*, a pixel x and a pair of offsets u and v, the feature is computed as

(1) $$F_{u,v}=D\left(x+\frac{u}{D(x)}\right)-D\left(x+\frac{v}{D(x)}\right)$$

By definition, $F_{u,v}$ is invariant with respect to in-plane translations and, due to the normalization by depth D(x), to depth variations. Furthermore, since few arithmetic and memory access operations are involved, it requires limited computational resources.

##### Training

The training set consists of segmented depthmap-labels pairs representing all the poses of interest for the application. An example of depthmap-labels pair is shown in Figure 1b,c. As in [23], we resort to a synthetic generator of the training set pairs by means of a 3D mesh model and a set of rendering routines: starting from a sub-set of representative hand poses, we extend the training set generating intermediate poses by means of key-frame animation.

The training phase aims at finding, for each node *n*, the most discriminative pair $\left(F,\tau \right)$, *i.e.*, the feature-threshold pair which maximizes the Gain of Information *I*:

(2) $$I=H\left(S_{n}\right)-\sum_{i\in L,R}\frac{|S_{i}|}{|S_{n}|}H\left(S_{i}\right)$$

where $S_{n}$ is the subset of pixels that reaches the current node, $S_{L}$ and $S_{R}$ are the two subsets obtained by the split against the threshold *τ*, and H(S) is the Shannon entropy with respect to classes for the subset S. After the best pair is chosen, the training procedure recourses on the left and right child nodes until a stopping criterion is met (e.g., maximum tree depth or minimum size of $S_{n}$). For each leaf node, the probability $P_{T}\left(c|x\right)$ is computed as the ratio between the number of pixels of class *c* and the total number of pixels that reach the leaf.

It is of relevance to note here that our experimental setup includes only two classes of movements and that the positioning of the hand with respect to the camera is highly constrained (see Section 2.1.3). This considerably reduces the set of possible hand poses, consequently reducing the size of the training set and the depth of the decision tree, drastically shortening the training time while still achieving satisfactory results. Furthermore, since the class of movement is known *a priori*, a single RF is trained for each specific exercise. Our custom implementation of the training algorithm that leverages on modern GPU architecture achieves a training time of around 40 h on a relatively inexpensive hardware configuration. The same training parameters set of [22] has been used (except for the tree depth that was set to 16).

##### Operator Hand Motion Estimation

The master unit consists of a commercial RGB-D camera (Softkinetic Depthsense DS325) that is suspended 50 cm over a table so that it can record operator’s hand movements from the top view. This placement is chosen to minimize the risk of self-occlusions among operator’s fingers, representing the main obstacle to the hand tracking task. The camera is linked to a laptop (Intel Core i7 3630QM, Nvidia GeForce 650M) running our custom implementation of the RF classifier. The laptop reads the depth input stream from the camera at a rate of 30 fps, which is the highest working frequency allowed by the camera. A preprocessing phase is devoted to isolate operator’s hand (foreground) from the table (background): pixels belonging to the plane (within 5 mm of uncertainty) are removed via the RANSAC algorithm [31], while the others are maintained for further processing. Once hand’s pixels have been segmented, their depth information is processed by the RF classifier which can recognize the 22 different parts of the hand. Then, the joints, fingertips, palm and wrist positions are approximated applying the Mean Shift clustering algorithm [32] on the hand sub-parts.

Only five of the total hand parts are kept for subsequent processing, as these are the only ones necessary for computing the master command signal, as explained in Section 2.1.3. Namely, these parts are the metacarpo-phalangeal (MCP), proximal- and distal-interphalangeal (PIP and DIP) joints and fingertip of the index, and the thumb fingertip [33].

#### 2.1.2. Slave Unit

**Figure 2:**
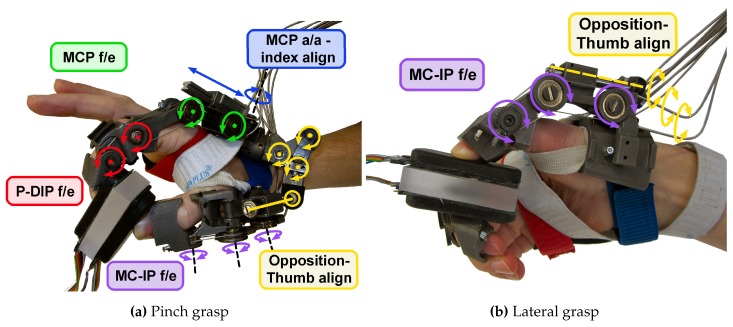
HX while holding the sensorized object in a pinch (**a**) and lateral (**b**) grasping exercise. The DoMs of the HX device are: (1) the flexion/extension of the index MCP; (2) of the index P-DIP (under-actuated); (3) of the thumb MCP and IP (under-actuated) and (4) the CMC opposition. Other Degrees-of-Freedom (DoF), like thumb intra/extra rotation and the index abduction/adduction, are passive [29]. The HX is used to grasp the sensorized object, whose squeezable soft-pads provide force information on the basis of a optoelectronic deformation transduction [34].

The slave unit consists of the HX powered hand orthosis [29], a mechatronic device built of three modules (Figure 2): a bi-digital wearable exoskeleton for the active assistance of the index and thumb fingers; a remote actuation block driving the exoskeleton by means of a cable-sheath system; and a control/power external unit. The exoskeleton is comprised of four active degrees-of-motion (DoM), two for each finger: for the index finger, they are the MCP joint, and the PIP and DIP joints, under-actuated together; for the thumb finger, they are the under-actuated flexion/extension (f/e) of the MCP and DIP joints, and the carpo-metacarpal (CMC) joint opposition. In the following, these four DoM are respectively addressed as MCP, P-DIP, MC-IP and CMC. DoM are driven by DC-motor, placed remotely in order to minimize the influence of weight and noise on the user, through a bidirectional cable-sheath transmission. Once worn and tethered to the actuators, the exoskeleton is not back-drivable, and it coerces motion on the wearer’s fingers. HX can drive each finger along the prescribed motion with a constant pressure of 20 N in tip, or equivalently distributed along the phalanges pads.

The slave unit also comprises a sensorized grasping object. It is a rectangular block (size 6 × 2.4 × 3 cm) of acrylic resin, with the widest faces covered by two pressure-sensitive pads, based on an opto-electronic sensing technology developed for measuring human-robot interaction forces in wearable rehabilitation robots [34]. Basically, the sensorized objects is grasped by squeezing two silicone bulk hollow structures (one per side of contact, see Figure 2). These pads cover a Printed Circuit Board (PCB) which hosts a pattern of pairs of light sensitive elements. Each pair includes an LED emitter and a light-sensitive receiver. When the silicone is squeezed, the deformation obstructs the light collected by the receiver with respect to the light emitted by the LED, and the proportional Voltage-drops of each receiver over the corresponding emitters is characterized in order to get the total normal displacement of the silicone pad. Previous work on the characterization of the sensor [30] reported an overall repeatability of the sensors (taking into account error and hysteresis) of about 0.16 N.

#### 2.1.3. Communication

The master and slave units are connected by means of a bidirectional communication link (UDP/IP connection). The communication protocol works at 30 fps rate, ensuring parallelism with master unit. The master unit encodes within a single byte per frame the data sent to the slave unit about the intended motor task, and it can receive feedbacks from the slave unit about (i) the kinematics and kinetic state of the exoskeleton and (ii) interaction forces with the sensorized object. A personal computer provides comprehensive RT information about both master and slave systems. Communication is engineered to require the lowest possible bandwidth. Our implementation showed good performances, since the delay between the master and the slave units was never more than 100 ms, and no data package was lost.

**Figure 3:**
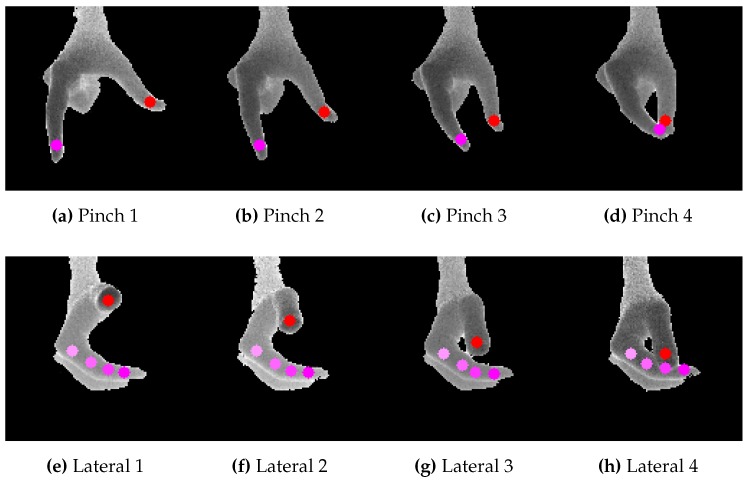
Pinch (**a–d**) and lateral (**e–h**) grasping sequences with overimposed fingertips as estimated by our VPE algorithm.

**Figure 4:**
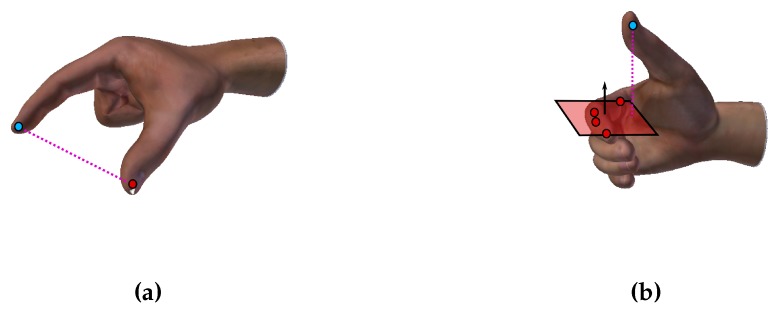
Graphical illustration of the distances computed to evaluate the percentage of completeness for the rehabilitative exercises: (**a**) pinch grasp; (**b**) lateral grasp. We used the 3D hand model from the libhand library [35] for illustration purpose.

To start the exercise, the operator has to place his hand under the camera. This constraint on the position is not representing a limitation to exercises, since it has been chosen to maximize both operator’s comfort (he can rest his elbow on the table, raising just his hand) and VPE accuracy (as fingers’ self-occlusions are minimized). Once his hand is acquired, the algorithm estimates hand’s joints of interest (example sequences are shown in Figure 3) and computes the percentage of completion of the exercise. For the pinch grasp, this percentage is related to the normalized distance between the index and thumb fingertips (see Figure 4a). For the lateral grasp, the percentage is related to the distance of thumb fingertip along the normal of the plane containing the index MCP, PIP, DIP joints and fingertip (see Figure 4b). These measures were chosen as they are very fast to compute.

### 2.2. Master–Slave Control Strategy

The closure percentage *p* is conveyed to the slave unit encoded in the one-byte payload of a UDP packet within seven bits, the remaining bit encoding the grasp type (0 for pinch, 1 for lateral grasping). The slave unit controller commands the exoskeleton motors according to the message received from the network, in order to reach the continuously updated desired position. According to the desired grasp, the four DoM are coordinated differently [20]: the set-point of the *i*-th DoM is commanded computing

(3) $$x_{i}=x_{i,0}+p\frac{x_{i,end}-x_{i,0}}{100}$$

where the maximum ($x_{i,end}$) and initial ($x_{i,0}$) values of the *i*-th joint opening $x_{i}$ are reported in Table 1.

**Table 1:** Reference values for hand exoskeleton (HX) actuator motions.

Grasp Type		MCP (deg)	P-DIP (deg)	MC-IP (deg)	CMC (deg)
Pinch	$x_{0}$	0	0	0	0
	$x_{end}$	90	60	45	75
Lateral	$x_{0}$	0	0	0	0
	$x_{end}$	75	100	65	45

### 2.3. Experimental Design and Methods

**Figure 5:**
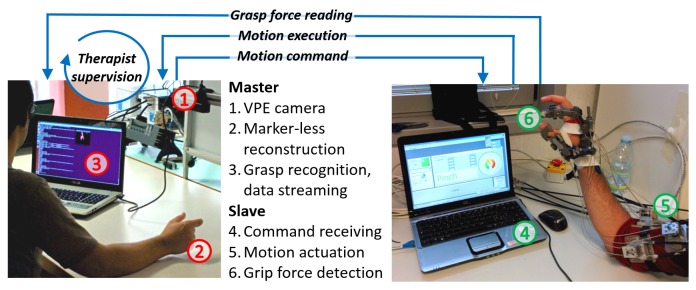
Our experimental setup provides an RT communication link, a natural direct driving of the gesture, and an on-the-fly supervision method of the operator.

The experimental protocol consists of repeated sequences of fingers “opening and closing” tasks, commanded by the operator of the master unit and executed on the subject’s hand by the slave exoskeleton. The experimental setup is illustrated in Figure 5.

In the experiments, we address two different kind of grasps, typically used within rehabilitative exercises, both involving only thumb and index fingers: the pinch and the lateral ones (illustrated in Figure 3). The operator chooses which exercise to perform before activating the RGB-D camera and starting acquiring images.

The experimental protocol comprises, for each subject, two series of repetitions for both pinch and lateral grasps at different speeds self-selected by the operator (30 repetitions per series, roughly divided as 10 each for “slow”, “normal” and “fast” velocities): the first series mainly aimed at letting the subjects familiarizing with the exoskeleton, while, in the second series, the subjects were asked to grasp the sensorized object while being guided in the rehabilitative exercises.

Within the exoskeleton, the *i*-th DoM motor of the slave setup tracked the $x_{i}$ set-points from Equation (3) according to a filtering stage (2nd-order low-pass Butterworth filter with cutoff frequency of 0.45 Hz) and a PI controller (estimated bandwidth of 80 Hz). As a consequence, the motor-driven positions—which we will indicate by $\hat{x}$—are delayed of 200 milliseconds *ca.*, however, being less noisy, with respect to the command signal $x_{i}$.

**Figure 6:**
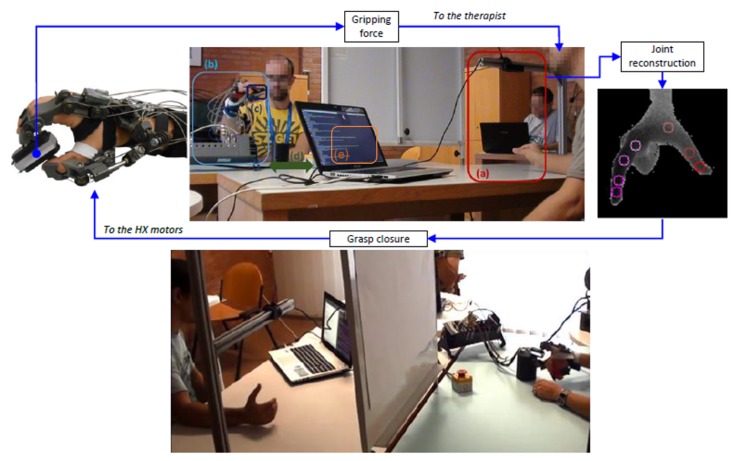
Experimental setup pipeline. (**a**) master unit: the VPE camera computes the joints positions of the operator’s hand; (**b**) slave unit: the subject wears the HX exoskeleton, which drives his/her fingers towards closure; the subject can freely move the arm; (**c**) squeezable grip sensor; (**d**) bi-directional link is realized through a UDP/IP communication between the VPE acquiring PC and the real-time control board driving the HX; (**e**) in the same PC, the operator visualizes in RT gripping force feedback from the slave unit. Down: same setup, addressing a lateral grasp. The white panel prevents the subject from having a visual clue of the operator’s intentions.

Since our research project is at its very first stage of development, we at the moment preferred to conduct an early stage validation of the system without directly including a real therapist nor any impaired (e.g., stroke survivors) subject, who will instead be involved in further and larger experiments. For this reason, we asked healthy right-handed subjects to volunteer for participate to this early validation stage. Six subjects participated in the experiments, while one volunteered to act as operator, and received training in VPE. The operator was acting as master in all the experiments. Subjects were introduced to the system and the protocol, and assisted in wearing the exoskeleton: they sat in front of the expert, but they had no visual cue of the operator’s intentions due to a panel. *Vice-versa*, the expert had visual feedback on the slave unit and received graphic feedback from both the hand tracker and the slave unit. An external PC was used for sniffing network traffic, data storing, postprocessing and statistics. The whole pipeline is depicted in Figure 6.

## 3. Results and Discussion

**Figure 7:**
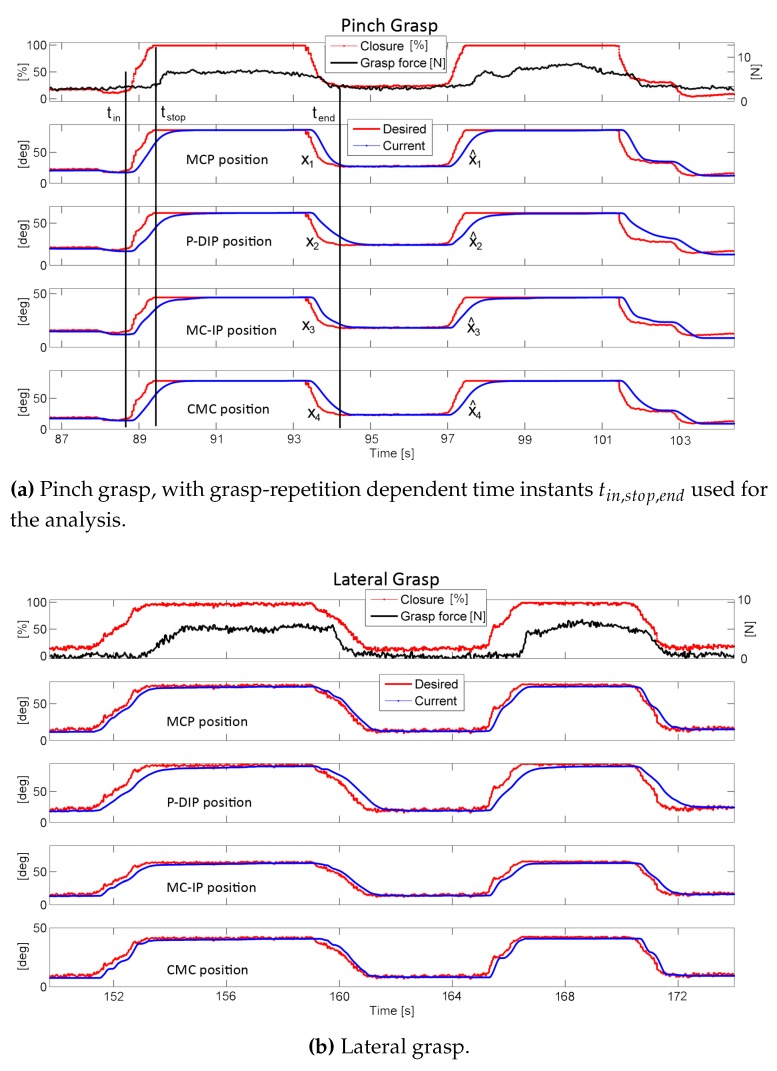
Example profiles of the tele-rehabilitation results. The first panel of (**a**) and (**b**) shows master desired (pre-filtering) closing percentage *p*, and grip force as recorded by the sensorized object, the other panels shows the slave desired (red) and measured (blue) position of the four DoM.

All six subjects could wear the exoskeleton without reporting any hindrance nor being harmed by the device, and the operator was able to correct and adapt the motion sequence based on the visual feedback of the patient’s environment. During the grasping session, the operator drove the closure of HX until the sensorized object was stably gripped: this condition could either be verified by the visual feedback, or by the interaction force reported by the sensor. Figure 7 shows illustrative trials of opening-closing sequences for both grasps. We analyzed the setup performance on the basis of the master input motion speed. Such speed was not set for each trial, since the operator was fully free to drive the motion; however, he was asked to try different speeds, based on his own perception. For each closure repetition, we estimated the speed from the slope of the closure command percentage—*i.e.*, the slope of the rising part of the dotted red curve in the first panel in Figure 7a,b.

To assess the performance of the slave setup in tracking the master commands, we analyzed the root-mean-squared error (RMSE) between the desired $x_{i}$ and the current motion of each DoM. Such performance would change as the operator varied the grasp velocity, due to the intrinsic limitation of the slave motors and the added inertia of the exoskeleton and of the mechanical transmission. Hence, we evaluated the distribution of the current-*versus*-desired motions discrepancy across the operator’s self-selected speed. Expected (but small) discrepancies between the motor-driven positions $\hat{x}$ and the command signal $x_{i}$ (respectively blue and red curves in the last panels of Figure 7a,b) are also due to the filtering stage described in Section 2.3.

For each grasp repetition, we isolated the motion profiles from just before the operator started closing ($t_{in}$) to just after the operator went back to open position ($t_{end}$), and we evaluated the RMSE *ε* in this time-window, while the closure speed $\dot{p}$ was evaluated as the mean slope in the closing phase (starting at $t_{in}$ and ending at $t_{stop}$):

(4) $$\varepsilon_{i}=\sqrt{\frac{\int_{t_{in}}^{t_{end}}{\left(x_{i}-{\hat{x}}_{i}\right)}^{2}dt}{t_{end}-t_{in}}}\;\text{with}\;i=1,\ldots ,4;\;\dot{p}=\frac{p\left(t_{stop}\right)-p\left(t_{in}\right)}{t_{stop}-t_{in}}\;$$

**Figure 8:**
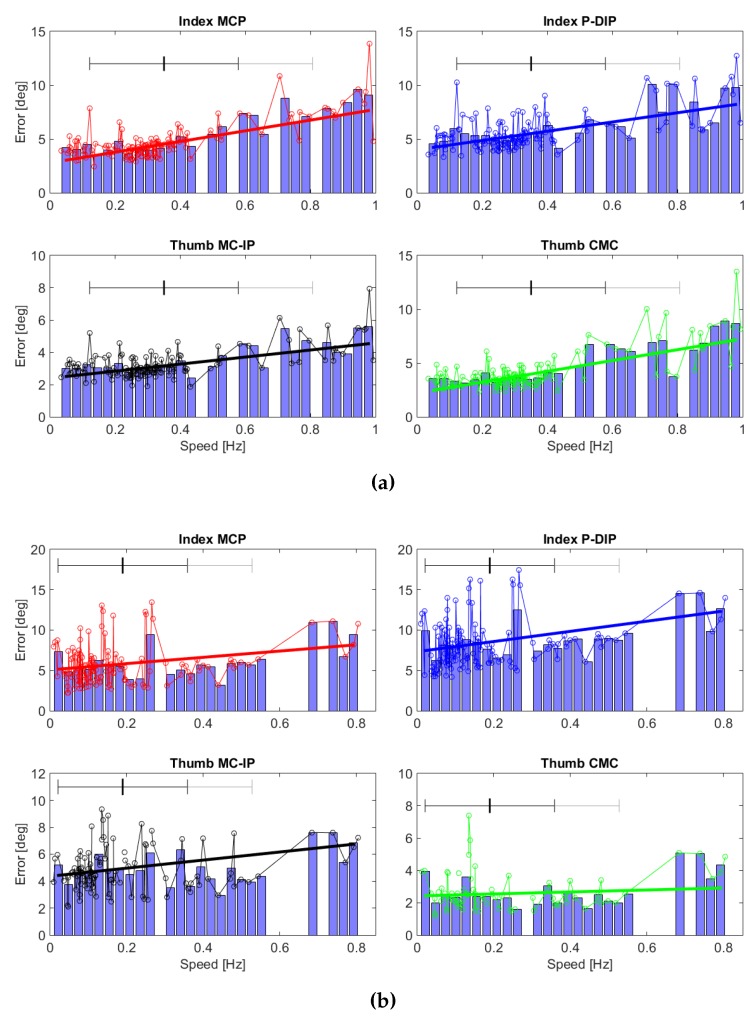
Aggregated cross-results of operator’s task execution speed and RMSE of HX motion from the desired, respectively for (**a**) pinch and (**b**) lateral grasps. The histogram bars represent the RMSE in degrees, while each task repetition is reported as a circle dot. The black small line in top of each histogram represents the mean speed, the black winds around it represent the standard deviation, and the gray wind indicates mean speed plus twice the standard deviation. Results are divided among the four DoM.

Aggregated results, comprising all subjects and all trials, separately per each DoM, are shown in Figure 8: each marker represents $\dot{p}$ and *ε* of a single closure trial. To proceed for a statistical analysis, we divided the operator speeds in 30 equal intervals, ranging from the minimum to the maximum $\dot{p}$ recorded. Within each speed interval, we estimated the mean value of the corresponding *ε* belonging to the second and the third quartile: such values are shown by the histograms in Figure 8. We interpolated these values with a linear function, weighting each mean *ε* with the number of closure trials enclosed in the correspondent speed interval: this linear relationship between $\dot{p}$ and *ε* is represented by the straight segments in Figure 8.

Results shown in Figure 7 demonstrate the instantaneous communication between the master command *p* (top panels, red curve) and the setpoint of the HX robot DoM $x_{i}$ (other panels, red solid curve), which are exactly aligned. For what concerns the motion actuation, the blue DoM output curve tracks the red input with a small delay and settling time, which are visually appreciable in the graphs but only cover 200 milliseconds *ca.*, and are due to the filtering and time-response of the actuators (see Section 2.3). On top of this, there is the communication delay between master and slave units, and another delay between the HX motion (blue curve) and the force response from the gripper (top black curve): this is the time needed by the HX to reach the gripper and squeeze it, the same effect is visible also in releasing the gripper. In any case, the delay between the operator reaching the desired posture and the peak response from the gripper was never noticeable in our experiments and never interfered with the exercises. Conversely, when the speed increases, the RMSE between the real and the desired motion, *ε*, also increases: this is visible from the plots in Figure 8. The main contribution to the calculated *ε* is mainly due to the discrepancy between $x_{i}$ and ${\hat{x}}_{i}$ in the grasping (increasing *p*) and releasing (decreasing *p*) dynamic phases, while in the *static* part the difference is not appreciable. Table 2 reports limit values (*Slow*, *Medium* and *Fast*) of the linear interpolation *ε* against closure speed $\dot{p}$ for each motor and each grasp type, and also the standard deviation from the collected data, if available: indeed, for certain speeds and especially in the lateral grasp, collected data were not enough to calculate a meaningful standard deviation.

A qualitative analysis of Figure 8 suggests that the operator preferred to concentrate grasping speeds in the 0÷0.85 Hz range for the pinch grasp, and in the 0÷0.5 Hz range for the lateral one (with 1 Hz representing a whole closure and opening task performed in one second). Quicker grasps that fall above these intervals, although being intercepted by linear regression, are still out of the statistics (being above mean speed plus twice the standard deviation). The *Medium* rows reported in Table 2 are chosen as the maximum speed value of these preferred range. In addition, we can notice how for the lateral grasp, *Medium* and *Fast* closure speeds attained are lower: this is mainly due to the fact that the HX motion covers a smaller space when *p* ranges from 0 to 100% (see Table 1).

**Table 2:** Mean value ± standard deviation of *ε*, classified according to the operator self-chosen velocity $\dot{p}$.

Grasp Type and Speed			MCP (deg)	P-DIP (deg)	MC-IP (deg)	CMC (deg)
Pinch	Slow	$\dot{p}<0.1$ Hz	3.0 ± 0.5	4.2 ± 1.0	2.5 ± 0.4	2.4 ± 0.6
	Medium	$\dot{p}\approx 0.35$ Hz	4.8 ± 1.7	5.7 ± 1.5	3.2 ± 0.8	4.2 ± 2.3
	Fast	$\dot{p}>0.85$ Hz	7.6 ± 3.7	8.2 ± 2.6	4.5 ± 1.8	7.1 ± 3.6
Lateral	Slow	$\dot{p}<0.05$ Hz	5.1 ± 2.1	7.4 ± 3.7	4.4 ± 1.1	2.4 ± 0.6
	Medium	$\dot{p}\approx 0.2$ Hz	6.1 ± 4.4	9.0 ± 5.7	5.1 ± 2.0	2.5 ± 1.0
	Fast	$\dot{p}>0.6$ Hz	8.1	12.1	6.7	2.8

Collected results demonstrate that the proposed experimental setup works reliably; in addition, low variances of the error *ε* shown in Table 2 demonstrate a good over-subject repeatability. In addition, they demonstrate that HX can actually drive the human hand along the imposed path and maintain the object grasp stably, that the hand tracking algorithm is capable of real-time performances and it is accurate enough for the purpose, and that the decoding algorithm of the master system is simple but effective, and does not require expensive materials (such as external sensors) nor an intensive phase of training. Furthermore, the operator could successfully drive the volunteers along the intended task in all trials, with any preferred speed setting, while being able to dynamically change it on-the-fly.

The proposed setup has been specifically studied to allow RT-direct telerehabilitation, with operator and patient simultaneously receiving mutual feedback. Still, the proposed setup could also be used in off-line rehabilitative tasks by recording the operator motion and commanding to the slave unit when requested (possibly multiple times): such a feature could be useful for patients who have to perform exactly the same exercise repeatedly. The operator could receive later a resume about patients’ performances.

Communication between master and slave units has been thought to require a very low bandwidth, thus allowing RT-direct controlled rehabilitation to take place even with unstable or poor Internet connection. No packet loss in the master–slave bidirectional communication was observed in our experiments, and exercises were never affected by appreciable communication delays. Delays will not in any case have consequences on the system stability, as the exoskeleton implements security mechanisms that prevents from harming the patient and will allow the exoskeleton to reach a rest position when no control command is received from the master unit; in case of delays, in addition, the operator could analyze off-line feedback received from the slave unit. The slave unit records information about patient range-of-motion for each addressed DoM as well as interaction forces. Such data can be used to assess improvements and patient’s evolution, representing useful and valid support both to the operator—who can prepare a set of rehabilitative exercises only once, thus saving time, and the patient—who can receive precise information about his/her improvements.

## 4. Conclusions

In this paper, we introduced the design of a telerehabilitation system for hand functional recovery, and presented the results of preliminary experimental activities assessing the system usability and accuracy.

The proposed system goes beyond the current state of the art in several features. In telerehabilitation systems, a strong limitation is usually due to time delays and loss of information [36], which might affect reliability and stability of the RT-link: the telecommunication system must comply with some minimum standards (maximum time lags, loss of information and speed). Our implementation showed good performances, since the lag between master and slave systems never affected the regular development of the exercises.

Most of the current rehabilitation robotic aids are independent mechanical systems and lowly networked, providing poor interaction with the operator. This represents a not negligible limitation because the operator needs to monitor patient’s progress to be able to adapt the exercises as needed. The main novelty of the presented application is given by the combination of a markerless hand tracking system, leveraging on a VPE algorithm, and the multi-joints HX hand exoskeleton. Together, they provide the operator with natural and reliable information about the evolution of the exercise kinematics and a way to enforce and change it. In addition, information from an external compliant sensor about interaction forces can be provided to the operator, to quantitatively evaluate whether the task’s goal was or not attained. Patients may feel more motivated to exercise at home under the guidance of an highly adaptive robotic tool.

Experimental results proved the overall feasibility, and the stability of the telerehabilitation setup across different speed settings, and for different subjects.

Future studies will deal with the extension of the VPE framework, to allow the automatic detection of the exercise accomplished by the operator. In addition, we will define clinical protocols to evaluate the efficacy of our telerehabilitation system with impaired subjects. In fact, hand rehabilitation therapy is relevant for post stroke patients, who often show residual hand functionality, which can be improved by continuous exercise. It is important to train patients constantly and effectively. Hemiplegic patients receive benefits from continuous exercise on the affected hand, especially if co-aided by the other hand they can still control. Post-traumatic healing and prevention of repeated injuries are as well achieved through rehabilitation. The aim of such treatment is to develop strength, flexibility, and proprioception in the affected body segment [37]. We think that our system paves the way to a set of telerehabilitation tools and procedures specifically designed for post stroke patients.

## Abbreviations

The following abbreviations are used in this manuscript:

a/a: abduction/adduction
f/e: flexion/extension
DIP: Distal-Intra-Phalangeal
DoM: Degree of Mobility
GPU: Graphics Processor Unit
HRI: Human Robot Interaction
HX: Hand eXoskeleton
MCP: Meta-Carpo-Phalangeal
MC-IP: Meta-Carpo-Inter-Phalangeal
P-DIP: Proximal-Distal-Intra-Phalangeal
PIP: Proximal-Intra-Phalangeal
PSO: Particle Swarm Optimization
RGB-D: Reg, Green, Blue, and Depth
RF: Random Rorest
RMSE: Root-Mean-Squared Error
RT: Real Time
UDP/IP: User Datagram Protocol/Internet Protocol
VPE: Vision-Based Pose Estimation
VR: Virtual Reality
